# Association between red cell distribution width-to-platelet ratio and short-term and long-term mortality risk in patients with acute ischemic stroke

**DOI:** 10.1186/s12883-023-03219-1

**Published:** 2023-05-15

**Authors:** Nan Xu, Cao Peng

**Affiliations:** 1 Department of Neurology, Jingzhou Hospital Affiliated to Yangtze University, Jingzhou, 434020 People’s Republic of China; 2 Department of Emergency, Union Hospital, Tongji Medical College, Huazhong University of Science and Technology, No. 1277, Jiefang Avenue, Wuhan, 430022 People’s Republic of China

**Keywords:** Red cell distribution width, Platelet, Ratio, Mortality risk, Acute ischemic stroke

## Abstract

**Background:**

The objective of this study was to evaluate the association between red cell distribution width/platelet ratio (RPR) and 30-day and 1-year mortality in acute ischemic stroke (AIS).

**Methods:**

Data for the retrospective cohort study were collected from the Medical Information Mart for Intensive Care (MIMIC) III database. RPR was divided into two groups: RPR ≤ 0.11 and RPR > 0.11. The study outcomes were 30-day mortality and 1-year mortality from AIS. Cox proportional hazard models were utilized to assess the association between RPR and mortality. Subgroup analyses were applied based on age, tissue-type plasminogen activator (IV-tPA), endovascular treatment, and myocardial infarction.

**Results:**

A total of 1,358 patients were included in the study. Short- and long-term mortality occurred in 375 (27.61%) and 560 (41.24%) AIS patients, respectively. A high RPR was significantly associated with increased 30-day [hazard ratio (HR): 1.45, 95% confidence interval (CI): 1.10 to 1.92, *P* = 0.009] and 1-year mortality (HR: 1.54, 95%CI: 1.23 to 1.93, *P* < 0.001) in AIS patients. Meanwhile, RPR was found to be significantly related to 30-day mortality in AIS patients aged < 65 years (HR: 2.19, 95% CI: 1.17 to 4.10, P = 0.014), without IV-tPA use (HR: 1.42, 95% CI: 1.05 to 1.90, *P* = 0.021), without using endovascular treatment (HR: 1.45, 95% CI: 1.08 to 1.94, *P* = 0.012), and without myocardial infarction (HR: 1.54, 95% CI: 1.13 to 2.10, *P* = 0.006). Additionally, RPR was associated with 1-year mortality in AIS patients aged < 65 years (HR: 2.54, 95% CI: 1.56 to 4.14, *P* < 0.001), aged ≥ 65 years (HR: 1.38, 95% CI: 1.06 to 1.19, *P* = 0.015), with (HR: 1.46, 95% CI: 1.15 to 1.85, *P* = 0.002) and without using IV-tPA (HR: 2.30, 95% CI: 1.03 to 5.11, *P* = 0.041), without using endovascular treatment (HR: 1.56, 95% CI: 1.23 to 1.96,* P* < 0.001), and without myocardial infarction (HR: 1.68, 95% CI: 1.31 to 2.15, *P* < 0.001).

**Conclusion:**

Elevated RPR is associated with a high risk of short-term and long-term mortality in AIS.

## Background

Stroke is one of the major causes of death and long-term disability, of which acute ischemic stroke (AIS) is the most common type, accounting for approximately 80% of all strokes [1, 2]. The 2020 American Heart Association report on Heart Disease and Stroke Statistics estimates that the prevalence of stroke in the United States in 2016 was 2.5%, corresponding to 7 million Americans over 20 years of age who had suffered a stroke, nearly 800,000 stroke events, and nearly 150,000 deaths [3]. AIS is a prominent cause of mortality, brings not only physical and mental suffering to the patients, but also placing a huge burden on families and society [4]. Therefore, it is essential to explore the factors associated with AIS and identify patients with poor prognoses for optimal management.

Strokes are caused by cerebral vascular occlusion or hemorrhage leading to oxygen and nutrient deprivation, which causes local inflammatory immune response [5]. Thereby, biochemical markers reflecting systemic immune inflammatory status may be of great significance for disease monitoring and prognosis in AIS patients. Several studies have explored and identified the overall association between serum inflammatory biomarkers and outcomes in patients with ischemic stroke [6–8]. A previous study observed that a higher admission systemic inflammatory response index (SIRI) increased the risk of poor outcome at 3 months in patients with AIS [8]. The red cell distribution width (RDW) is a parameter reflecting the variation and dispersion degree of peripheral blood red blood cell volume [9] and is clinically used to distinguish different types of anemia [10]. In recent years, RDW has attracted attention as an inflammatory marker of cardiovascular and cerebrovascular diseases [11]. A cohort study and systematic review reported that RDW is associated with mortality after AIS [12]. The results of a previous study implied that pre-RDW is a reliable predictor of one-year prognosis and mortality after receiving endovascular therapy in acute anterior circulation stroke patients [13]. Platelets attach to the damaged blood vessel wall, release bioactive substances, and promote the formation of thrombosis [14]. Platelets have been found to be predictors of prognosis in cerebral infarction and cerebral infarction [15]. A previous study showed that platelet aggregation-related thrombosis is a key step in the pathophysiology of AIS [16]. RDW to platelet ratio (RPR) is a new marker of inflammation, which has been found to be related to the prognosis of cerebrovascular diseases associated with inflammation such as cerebral hemorrhage and acute myocardial infarction [17, 18]. However, the association of RPR with AIS outcome has not been reported.

Therefore, the aim of this study was to explore the association between RPR and short-term and long-term mortality in patients with AIS and help to evaluate its predictive value for mortality in order to screen out a powerful marker for risk stratification of AIS in the future.

## Methods

### Study design and patients

This was a retrospective cohort study. We derived data from the Medical Information Mart for Intensive Care-III (MIMIC-III) database with intensive care unit (ICU) admission diagnoses classified as ICD-9 code: 43,301, 43,311, 43,321, 43,331, 43,381, 43,391, 43,401, 43,411, 43,491. MIMIC-III is a large, free accessible intensive care database that contains the detailed information of more than 40,000 patients admitted to the critical care units in the Beth Israel Deaconess Medical Center (BIDMC) from 2001 to 2012, including demographic data, vital signs, comorbidities, and laboratory tests, which provides reliable data resource for clinicians to conduct epidemiological studies. The included criteria were (1) diagnosed as AIS at ICU admission. Excluded criteria were (1) aged < 18 years old; (2) hospitalized in the ICU for less than 24 h and (3) missing key data, such as RDW, and platelet. Our study data were obtained from a public database and therefore did not require approval from the ethics committee of our hospital.

### Data collection

We recorded patients’ information including: (1) baseline characteristics: age (years), gender, ethnicity (Black, White, others, and unknown), insurance status (Medicare, private, and others), therapy use [ventilation use (yes or no), vasopressor use (yes or no), renal replacement therapy (Rrt, yes or no), tissue-type plasminogen activator (IV-tPA, yes or no), endovascular treatment (yes or no), Beta-blockers (yes or no)], comorbidities (diabetes, congestive heart failure, atrial fibrillation, acute myocardial infarction, hypertension, cardiogenic shock, acute kidney injury, and myocardial infarct), weight (kg); (2) vital signs: systolic blood pressure (SBP, mmHg), diastolic blood pressure (DBP, mmHg), heart rate (bpm), respiratory rate (bpm), temperature (°C), SpO_2_ (%); (3) scoring systems: sequential organ failure assessment (SOFA) score, simplified acute physiology score II (SAPSII), quick sepsis-related organ failure assessment (qSOFA) score, Glasgow Coma Scale (GCS), charlson’s comorbidity index (CCI), international normalized ratio (INR); (4) laboratory parameters: white blood cell (WBC) count (K/uL), platelet (K/uL), hemoglobin (g/dL), RDW (10^9^/L), creatinine (mg/dL), prothrombin time (PT, seconds), blood urea nitrogen (BUN, mg/dL), glucose (mg/dL), sodium (mEq/L), potassium (mEq/L), chloride (mEq/L).

### Definitions and outcomes

The RPR is defined using the following equation: RDW/platelet count. According to the cut-off value, RPR was divided into groups: RPR ≤ 0.11 and RPR > 0.11. The outcome of the study was 30-day mortality and 1-year mortality, which were defined as the time from ICU admission to death or the last date of follow-up. The follow-up duration was 30.00 (22.05, 30.00) days for the 30-day mortality outcome and 365.00 (22.05, 365.00) days for the 1-year mortality outcome.

### Statistical analysis

The measurement data with normal distribution were described by Mean ± standard deviation (Mean ± SD), and the t-test was used to compare the differences between the two groups. The median and quartile [M (Q_1_, Q_3_)] were used to describe the distribution of measurement data that did not obey the normal distribution, and the Wilcoxon rank sum test was used to compare the differences between the two groups. The number of cases and constituent ratio [n (%)] was used to describe the distribution of count data, and the chi-square test was used to compare the differences between groups.

Univariate Cox proportional-hazards models were used to explore the effect of all variables on 30-day and 1-year mortality to screen for confounders. The significant variables in univariate analysis were screened one by one by stepwise regression method for multivariate analysis. Model 1 was an unadjusted model, in Model 2 of 30-day mortality, age, ethnicity, ventilation, respiratory rate, SAPSII, SOFA, and glucose were adjusted for; age, ethnicity, ventilation, respiratory rate, heart rate, SAPSII, SOFA, creatinine, and glucose were adjusted in model 2 for 1-year mortality.

The random forest imputation method was used to impute data for variables with missing rates of less than 20%. Sensitivity analysis was performed by comparing the data before and after the imputation. Subgroup analyses were applied based on age, IV-tPA, endovascular treatment, and myocardial infarction. Kaplan–Meier (KM) survival analysis was used to assess and compare the disease-related survival of patients with different RPR classifications. The hazard ratio (HR) with a 95% confidence interval (CI) was used to assess the results. X-tile Version 3.6.1 software was used to determine the best cut-off value for RPR classification. We also used restricted cubic spline (RCS) transformations to assess nonlinear relationships between RPR and mortality risk in patients with AIS. The significance for all tests was set at an alpha level of 0.05. Statistical analyses were performed using SAS 9.4 (SAS Institute Inc., Cary, NC, USA), Python 3.7.4, and R version 4.2.0 (2022–04-22 ucrt).

## Results

### Baseline characteristics of included patients

**Fig. 1:**
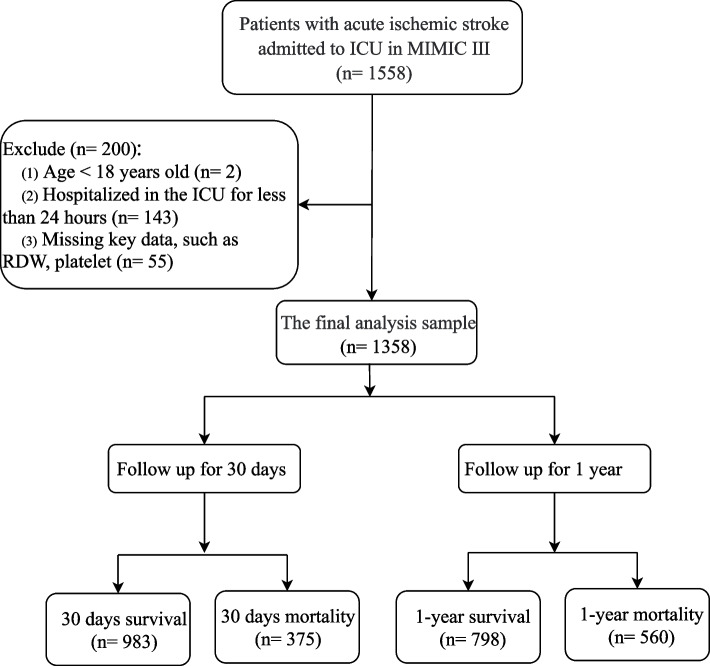
The flow chart of data collection

A loss to follow-up rate of 0% was estimated. Based on the inclusion and exclusion criteria, 1,358 patients were included in the study, of which 375 patients (27.61%) had 30-day mortality, and 560 patients (41.24%) ended up with 1-year mortality. The flow chart of data collection is provided in Fig. 1. The mean age of the patients was 68.76 ± 15.60 years. The majority of the included population was White (72.16%). There were significant differences between patients with RPR ≤ 0.11 and patients with RPR > 0.11 in ventilation, vasopressor, Rrt, IV-tPA, SBP, DBP, heart rate, SAPSII, SOFA, qSOFA, CCI, INR, diabetes, atrial fibrillation, WBC, platelet, hemoglobin, RDW, creatinine, PT, BUN, and chloride. Baseline demographic and clinical characteristics and laboratory tests of the included patients are shown in Table 1.

**Table 1:** Baseline characteristics of included patients

Variables	Total (*n* = 1358)	RPR ≤ 0.11 (*n* = 1176)	RPR > 0.11 (*n* = 182)	Statistics	*P*
Age, years, Mean ± SD	68.76 ± 15.60	68.56 ± 15.86	70.08 ± 13.77	t = -1.36	0.175
Gender, n (%)				χ^2^ = 0.122	0.726
Female	685 (50.44)	591 (50.26)	94 (51.65)		
Male	673 (49.56)	585 (49.74)	88 (48.35)		
Ethnicity, n (%)				χ^2^ = 2.020	0.568
Black	121 (8.91)	104 (8.84)	17 (9.34)		
Others	164 (12.08)	138 (11.73)	26 (14.29)		
Unknown	93 (6.85)	84 (7.14)	9 (4.95)		
White	980 (72.16)	850 (72.28)	130 (71.43)		
Insurance, n (%)				χ^2^ = 0.759	0.684
Medicare	848 (62.44)	733 (62.33)	115 (63.19)		
Others	136 (10.01)	121 (10.29)	15 (8.24)		
Private	374 (27.54)	322 (27.38)	52 (28.57)		
Ventilation, n (%)				χ^2^ = 23.297	< 0.001
No	636 (46.83)	581 (49.40)	55 (30.22)		
Yes	722 (53.17)	595 (50.60)	127 (69.78)		
Vasopressor, n (%)				χ^2^ = 35.141	< 0.001
No	884 (65.10)	801 (68.11)	83 (45.60)		
Yes	474 (34.90)	375 (31.89)	99 (54.40)		
Rrt, n (%)				χ^2^ = 13.020	< 0.001
No	1285 (94.62)	1123 (95.49)	162 (89.01)		
Yes	73 (5.38)	53 (4.51)	20 (10.99)		
Iv-tPA, n (%)				χ^2^ = 20.859	< 0.001
No	1121 (82.55)	949 (80.70)	172 (94.51)		
Yes	237 (17.45)	227 (19.30)	10 (5.49)		
Beta blockers, n (%)				χ^2^ = 1.275	0.259
No	1264 (93.08)	1091 (92.77)	173 (95.05)		
Yes	94 (6.92)	85 (7.23)	9 (4.95)		
Endovascular treatment, n (%)				χ^2^ = 2.627	0.105
No	1268 (93.37)	1093 (92.94)	175 (96.15)		
Yes	90 (6.63)	83 (7.06)	7 (3.85)		
Weight, kg, Mean ± SD	78.17 ± 17.85	78.27 ± 17.70	77.55 ± 18.82	t = 0.50	0.616
SBP, mmHg, Mean ± SD	138.54 ± 28.30	140.81 ± 28.27	123.89 ± 23.80	t = 8.69	< 0.001
DBP, mmHg, Mean ± SD	69.88 ± 18.32	70.46 ± 18.37	66.17 ± 17.56	t = 2.95	0.003
Respiratory rate, bpm, Mean ± SD	18.26 ± 5.18	18.15 ± 5.08	18.95 ± 5.75	t = -1.76	0.079
Heart rate, bpm, Mean ± SD	83.72 ± 18.97	82.93 ± 18.64	88.82 ± 20.30	t = -3.91	< 0.001
Temperature, °C, Mean ± SD	36.61 ± 0.95	36.62 ± 0.90	36.55 ± 1.23	t = 0.68	0.494
SpO2, %, Mean ± SD	98.02 ± 2.40	98.01 ± 2.41	98.09 ± 2.32	t = -0.43	0.667
SAPSII, score, M (Q_1_, Q_3_)	35.00 (27.00, 45.00)	34.00 (26.00, 43.00)	44.00 (35.00, 54.00)	Z = 8.324	< 0.001
SOFA, score, M (Q_1_, Q_3_)	3.00 (2.00, 6.00)	3.00 (2.00, 5.00)	6.00 (4.00, 9.00)	Z = 12.395	< 0.001
qSOFA, score, M (Q_1_, Q_3_)	2.00 (1.00, 2.00)	2.00 (1.00, 2.00)	2.00 (1.00, 2.00)	Z = 1.969	0.049
GCS, score, Mean ± SD	12.53 ± 3.34	12.54 ± 3.26	12.42 ± 3.80	t = 0.41	0.679
CCI, M (Q_1_, Q_3_)	6.00 (5.00, 8.00)	6.00 (5.00, 8.00)	7.00 (5.00, 9.00)	Z = 4.064	< 0.001
INR, ratio, M (Q_1_, Q_3_)	1.22 (1.10, 1.40)	1.20 (1.10, 1.30)	1.40 (1.20, 1.80)	Z = 8.418	< 0.001
Diabetes, n (%)				χ^2^ = 8.335	0.004
No	448 (32.99)	405 (34.44)	43 (23.63)		
Yes	910 (67.01)	771 (65.56)	139 (76.37)		
Congestive heart failure, n (%)				χ^2^ = 0.934	0.334
No	959 (70.62)	836 (71.09)	123 (67.58)		
Yes	399 (29.38)	340 (28.91)	59 (32.42)		
Atrial fibrillation, n (%)				χ^2^ = 6.051	0.014
No	1004 (73.93)	883 (75.09)	121 (66.48)		
Yes	354 (26.07)	293 (24.91)	61 (33.52)		
Acute myocardial infarction, n (%)				χ^2^ = 0.455	0.500
No	718 (52.87)	626 (53.23)	92 (50.55)		
Yes	640 (47.13)	550 (46.77)	90 (49.45)		
Hypertension, n (%)				χ^2^ = 0.085	0.770
No	1200 (88.37)	1038 (88.27)	162 (89.01)		
Yes	158 (11.63)	138 (11.73)	20 (10.99)		
Cardiogenic shock, n (%)				χ^2^ = 0.260	0.610
No	455 (33.51)	391 (33.25)	64 (35.16)		
Yes	903 (66.49)	785 (66.75)	118 (64.84)		
Acute kidney injury, n (%)				-	1.000
No	1333 (98.16)	1154 (98.13)	179 (98.35)		
Yes	25 (1.84)	22 (1.87)	3 (1.65)		
Myocardial infarct, n (%)				χ^2^ = 0.616	0.432
No	1129 (83.14)	974 (82.82)	155 (85.16)		
Yes	229 (16.86)	202 (17.18)	27 (14.84)		
WBC, K/uL, M (Q_1_, Q_3_)	11.10 (8.30, 14.20)	11.20 (8.60, 14.10)	9.80 (6.40, 14.70)	Z = -2.871	0.004
Platelet, K/uL, M (Q_1_, Q_3_)	215.00 (163.00, 278.00)	232.00 (186.00, 288.00)	101.00 (66.00, 121.00)	Z = -21.420	< 0.001
Hemoglobin, g/dL, Mean ± SD	11.54 ± 2.12	11.79 ± 2.02	9.92 ± 2.08	t = 11.63	< 0.001
RDW, %, Mean ± SD	14.53 ± 1.80	14.31 ± 1.58	16.02 ± 2.36	t = -9.45	< 0.001
Creatinine, mg/dL, M (Q_1_, Q_3_)	0.90 (0.70, 1.20)	0.90 (0.70, 1.20)	1.00 (0.70, 1.50)	Z = 3.229	0.001
PT, seconds, M (Q_1_, Q_3_)	13.72 (13.10, 15.20)	13.69 (13.00, 14.70)	15.25 (13.70, 17.90)	Z = 8.456	< 0.001
BUN, mg/dL, M (Q_1_, Q_3_)	18.00 (13.00, 26.00)	17.00 (13.00, 25.00)	23.00 (14.00, 35.00)	Z = 5.204	< 0.001
Glucose, mg/dL, M (Q_1_, Q_3_)	131.00 (108.00, 165.00)	131.00 (108.00, 164.00)	139.50 (113.00, 178.00)	Z = 1.947	0.052
Sodium, mEq/L, Mean ± SD	139.14 ± 4.37	139.16 ± 4.31	139.01 ± 4.75	t = 0.45	0.655
Potassium, mEq/L, Mean ± SD	4.04 ± 0.71	4.03 ± 0.71	4.10 ± 0.76	t = -1.09	0.275
Chloride, mEq/L, Mean ± SD	105.64 ± 5.37	105.41 ± 5.13	107.14 ± 6.54	t = -3.43	< 0.001
LOS, days, M (Q_1_, Q_3_)	3.61 (1.96, 7.86)	3.23 (1.90, 7.06)	6.17 (2.99, 11.37)	Z = 6.462	< 0.001
Outcome, n (%)				χ^2^ = 18.819	< 0.001
Death	1038 (76.44)	922 (78.40)	116 (63.74)		
Survival	320 (23.56)	254 (21.60)	66 (36.26)		
30-day stay, days, M (Q_1_, Q_3_)	30.00 (22.05, 30.00)	30.00 (24.62, 30.00)	30.00 (10.14, 30.00)	Z = -3.237	0.001
30-day outcome, n (%)				χ^2^ = 12.372	< 0.001
Death	983 (72.39)	871 (74.06)	112 (61.54)		
Survival	375 (27.61)	305 (25.94)	70 (38.46)		
1-year stay, days, M (Q_1_, Q_3_)	365.00 (22.05, 365.00)	365.00 (24.62, 365.00)	116.08 (10.14, 365.00)	Z = -5.158	< 0.001
1-year outcome, n (%)				χ^2^ = 35.746	< 0.001
Death	798 (58.76)	728 (61.90)	70 (38.46)		
Survival	560 (41.24)	448 (38.10)	112 (61.54)		

*t* t-test, *Z* wilcoxon rank sum test, *χ*^*2*^ chi-square test, *SD* standard deviation, *M* Median, *Q*_*1*_ 1st Quartile, *Q*_*3*_ 3st Quartile, *RPR* red cell distribution width (RDW) to platelet ratio, *Rrt* renal replacement therapy, *Iv-tPA* tissue-type plasminogen activator, *SBP* systolic blood pressure, *DBP* diastolic blood pressure, *SAPSII* simplified acute physiology score II, *SOFA* sequential organ failure assessment, *qSOFA* quick sepsis-related organ failure assessment, *GCS* Glasgow Coma Scale, *CCI* charlson’s comorbidity index, *INR* international normalized ratio, *WBC* white blood cell, *PT* prothrombin time, *BUN* blood urea nitrogen, *LOS* length of stay

### Association of RPR with 30-day and 1-year mortality in patients with AIS

In model 1 analysis, RPR > 0.11 was associated with 30-day mortality (HR: 1.58, 95% CI: 1.22 to 2.04, *P* < 0.001) and 1-year mortality (HR: 1.85, 95% CI: 1.50 to 2.28,* P* < 0.001) in patients with AIS. Based on model 2 analysis, RPR > 0.11 was associated with increased risk of 30-day (HR: 1.45, 95% CI: 1.10 to 1.92, *P* = 0.009) and 1-year mortality (HR: 1.54, 95% CI: 1.23 to 1.93, *P* < 0.001) in AIS patients. The association of RPR with 30-day and 1-year mortality in patients with AIS is presented in Table 2.

**Table 2:** Association of RPR with 30-day and 1-year mortality in patients with AIS

Variables	Model 1		Model 2	
	HR (95% CI)	*P*	HR (95% CI)	*P*
30-day mortality				
RPR				
≤ 0.11	Ref		Ref	
> 0.11	1.58 (1.22–2.04)	< 0.001	1.45 (1.10–1.92)	0.009
1-year mortality				
RPR				
≤ 0.11	Ref		Ref	
> 0.11	1.85 (1.50–2.28)	< 0.001	1.54 (1.23–1.93)	< 0.001

Notes: *Ref* reference, *HR* hazard ratio, *CI* confidence interval, *RPR* red cell distribution width (RDW) to platelet ratio

Model 1: unadjusted mode; Model 2 of 30-day mortality adjusted for age, ethnicity, ventilation, respiratory rate, SAPSII, SOFA, and glucose; Model 2 of 1-year mortality adjusted for age, ethnicity, ventilation, respiratory rate, heart rate, SAPSII, SOFA, creatinine, and glucose

**Fig. 2:**
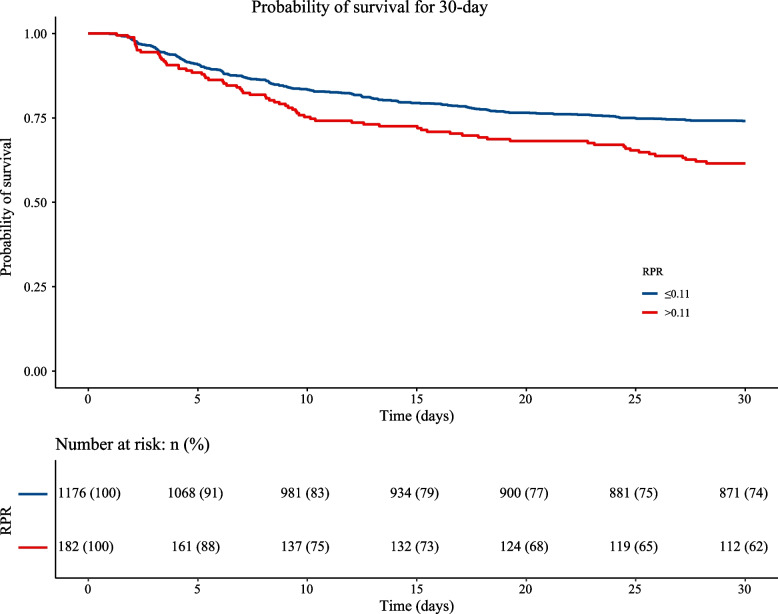
KM curve of 30-day mortality in AIS

**Fig. 3:**
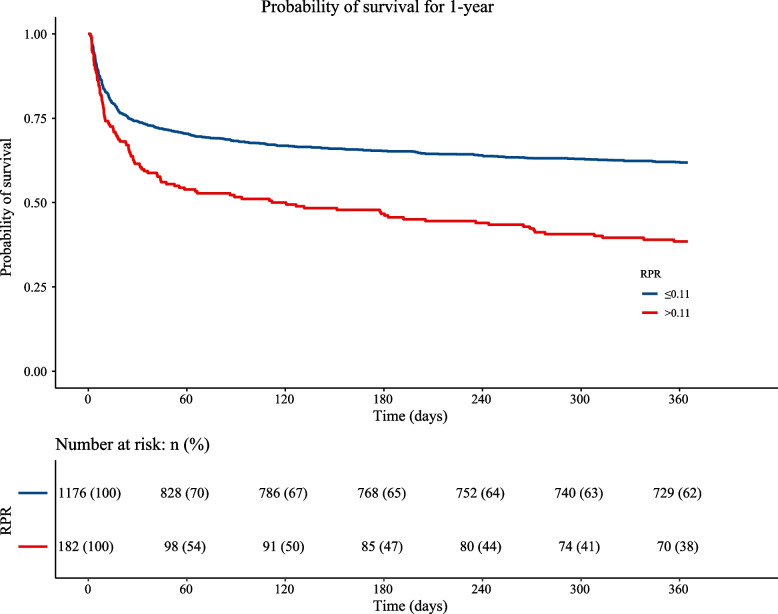
KM curve of 1-year mortality in AIS

**Fig. 4:**
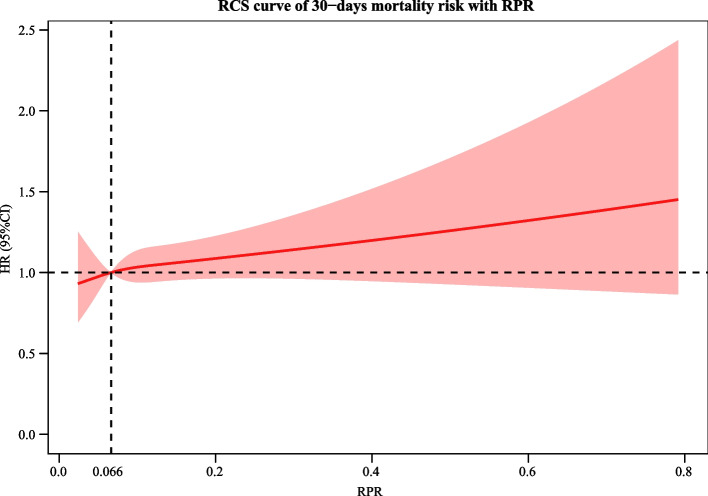
The association between RPR and 30-day in patients with AIS based on the RCS analysis

**Fig. 5:**
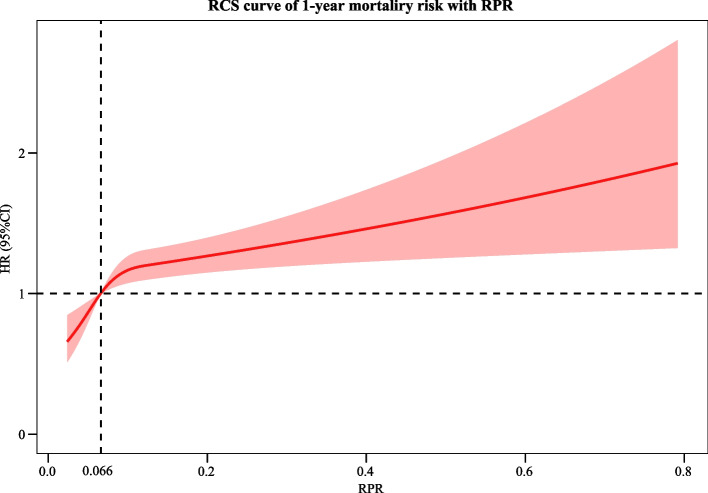
The association between RPR and 1-year mortality in patients with AIS based on the RCS analysis

The KM curve shows a higher risk of 30-day and 1-year mortality in patients with an RPR > 0.11. The KM curve for 30-day mortality in AIS is depicted in Fig. 2. The KM curve of 1-year mortality in AIS is shown in Fig. 3. RCS curves show a nonlinear association between RPR and 30-day and 1-year mortality in patients with AIS. The association between RPR and 30-day and 1-year mortality in patients with AIS based on the RCS analysis is shown in Figs. 4 and 5.

### Subgroup analyses of the associations of RPR with 30-day and 1-year mortality in patients with AIS

**Fig. 6:**
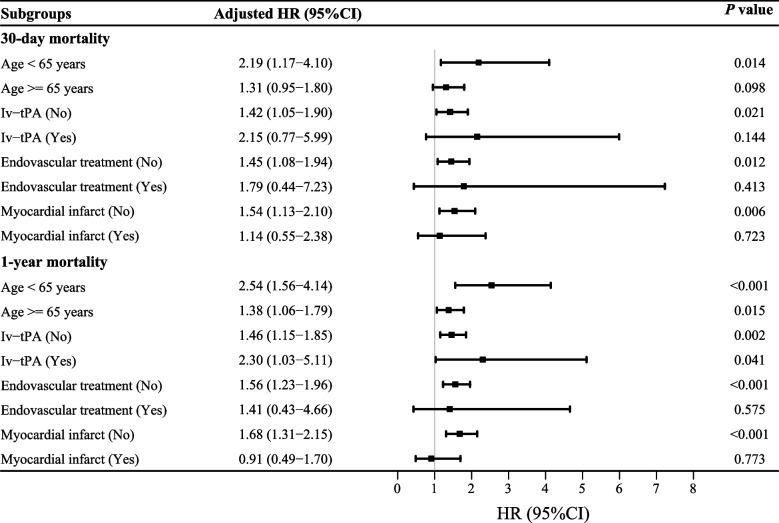
Subgroup analyses of the associations of RPR with 30-day and 1-year mortality in patients with AIS

Subgroup analyses based on age, IV-tPA, endovascular treatment, and myocardial infarction were performed to further investigate the association of RPR with 30-day and 1-year mortality in different stratifications. Regarding the outcome of 30-day mortality, RPR was associated with 30-day mortality in AIS patients aged < 65 years (HR: 2.19, 95% CI: 1.17 to 4.10, *P* = 0.014), in AIS patients without using IV-tPA (HR: 1.42, 95% CI: 1.05 to 1.90, *P* = 0.021), in AIS patients without using endovascular treatment (HR: 1.45, 95% CI: 1.08 to 1.94, *P* = 0.012), and in AIS patients without myocardial infarction (HR: 1.54, 95% CI: 1.13 to 2.10, *P* = 0.006). Regarding the 1-year mortality in patients with AIS, RPR was associated with 1-year mortality in AIS patients with age < 65 years old (HR: 2.54, 95% CI: 1.56 to 4.14, *P* < 0.001), age ≥ 65 years old (HR: 1.38, 95% CI: 1.06 to 1.19, *P* = 0.015), in AIS patients with (HR: 1.46, 95% CI: 1.15 to 1.85,* P* = 0.002) and without using IV-tPA (HR: 2.30, 95% CI: 1.03 to 5.11,* P* = 0.041), in AIS patients without using endovascular treatment (HR: 1.56, 95% CI: 1.23 to 1.96, *P* < 0.001), and in AIS patients without myocardial infarction (HR: 1.68, 95% CI: 1.31 to 2.15, *P* < 0.001). Subgroup analyses of the associations of RPR with 30-day and 1-year mortality in patients with AIS are shown in Fig. 6.

## Discussion

In recent years, numerous studies have focused on the role of inflammatory processes in the aetiology and prognosis of AIS [19, 20]. Thus, in this study, we explored the association between RPR and short-term and long-term mortality in patients with AIS. The results of the current study revealed that there was a nonlinear relationship between RPR and mortality in AIS patients, with an upward trend. Elevated RPR was significantly linked to increased 30-day mortality and 1-year mortality in AIS. RPR was also related to 30-day mortality in AIS patients younger than 65 years old, without the use of IV-tPA, without the use of endovascular treatment, and without myocardial infarction. RPR was associated with 1-year mortality in AIS patients aged < 65 years, aged ≥ 65 years, with and without IV-tPA, without endovascular therapy, and without myocardial infarction.

Based on RDW and platelets, RPR has served as a useful systemic inflammatory marker and prognostic indicator of adult inflammatory diseases such as hepatic fibrosis in chronic hepatitis B [21], myocardial infarction [22], and acute pancreatitis [23]. To our knowledge, there are no reports on RPR in mortality in AIS. Our results indicated the association between RPR and short-term and long-term mortality risk in patients with AIS. The roles of RDW and platelet in the motility of AIS may be the underlying mechanisms. An elevated RDW level denotes an aberrant variation of red blood cell size in the peripheral blood [24], which is predisposed to thrombophilia due to increased or ineffective red blood cells production and excessive fragmentation or destruction of red blood cell [25]. RDW has been studied as an inflammatory marker in stroke severity [26]. Previous studies have also revealed that RDW is associated with the occurrence and prognosis of AIS [27–29]. A study by Feng et al. revealed higher RDW levels were related to worsening prognosis in AIS patients [30]. Platelets serve as a link between innate and acquired immune responses and contribute to the beginning or exacerbation of the inflammatory process by secreting proinflammatory cytokines and interacting with a variety of immune cells, such as neutrophils, T-lymphocytes, natural killer cells, and macrophages [31]. AIS may cause aberrant platelet function, and activated and hyperresponsive platelets may interact with platelet-binding T lymphocyte cells to create cytokines, interferons, chemokines, and other adhesion molecules that may interfere with AIS recovery [32].

According to the subgroup analysis, the association between high RPR and mortality became non-significant in the subgroups with the IV-tPA use, the endovascular therapy use, and myocardial infarction, however, this association remained significant in the AIS patients without the use of IV-tPA, without the use of endovascular treatment, and without myocardial infarction. We hypothesized that there might be an interaction between RPR and disease severity. Patients with IV-tPA use, endovascular therapy use, and myocardial infarction, to a certain degree, represented patients with inflammation of higher severity. Additionally, treatment of AIS may affect the outcome of AIS. Early administration of IV-tPA and/or endovascular therapy has been found to be associated with improved outcomes in AIS [33]. However, Longstreth et al. indicate that 3-month functional outcomes after an AIS were not significantly associated with IV-tPA use [34]. Similarly, AIS patients with myocardial infarction may differ from AIS without myocardial infarction. Inohara et al. found that among older AIS patients treated with recombinant tissue-type plasminogen activator (rtPA), recent myocardial infarction was associated with an increased risk of in-hospital mortality [35]. Further research is required to confirm the link between RPR and mortality in AIS patients receiving therapy or with myocardial infarction.

In this study, the association between RPR and short- and long-term survival in patients with AIS was confirmed using rigorous statistical methods, which may help in the monitoring and risk stratification management of AIS. Furthermore, in clinical practice, RDW and platelets are commonly used to measure whole blood counts, which can be easily monitored dynamically due to the speed and low cost of the method. There are several limitations to this study that should be considered. Firstly, this study is a retrospective, single-center study design, which inevitably introduces some bias. Second, although the patients included were multiracial, this study used data from ICUs in the United States, and the results may not be generalizable to ICUs in other countries. Third, long-term mortality is difficult to adjust for confounding factors, such as out-of-hospital interventions, and should be interpreted with caution. Fourth, increased inflammatory biomarkers (C-reactive protein and erythrocyte sedimentation rate) have been shown to increase poor outcomes in ischemic stroke. However, C-reactive protein and erythrocyte sedimentation rate data are missing from the database. Further studies are needed to assess the association between RPR and 30-day and 1-year mortality in AIS, with C-reactive protein and erythrocyte sedimentation rate being considered.

## Conclusions

In conclusion, higher RPR was correlated with higher 30-day and 1-year mortality in AIS patients, especially in AIS patients without IV-tPA use, without endovascular therapy, and without myocardial infarction.

## Data Availability

The datasets generated and/or analyzed during the current study are available in the MIMIC-III database, https://mimic.physionet.org/iii/.

## Abbreviations

AIS: Acute ischemic stroke
SIRI: Systemic inflammatory response index
RDW: Red cell distribution width
RPR: RDW to platelet ratio
MIMIC-III: Medical Information Mart for Intensive Care-III
ICU: Intensive care unit
BIDMC: Beth Israel Deaconess Medical Center
SBP: Systolic blood pressure
DBP: Diastolic blood pressure
SOFA: Sequential organ failure assessment
SAPSII: Simplified acute physiology score II
qSOFA: Quick sepsis-related organ failure assessment
GCS: Glasgow Coma Scale
CCI: Charlson’s comorbidity index
INR: International normalized ratio
WBC: White blood cell
PT: Prothrombin time
BUN: Blood urea nitrogen
